# Is changing status through housing tenure associated with changes in mental health? Results from the British Household Panel Survey

**DOI:** 10.1136/jech-2014-203990

**Published:** 2014-10-07

**Authors:** Frank Popham, Lee Williamson, Elise Whitley

**Affiliations:** 1 MRC/CSO Social and Public Health Sciences Unit, University of Glasgow, Glasgow, UK; 2 Institute of Geography, University of Edinburgh, Edinburgh, UK

**Keywords:** MENTAL HEALTH, HOUSING, INEQUALITIES

## Abstract

**Background:**

Actual or perceived status, such as housing tenure, may impact on health through stress-inducing social comparisons. Studies of how status change impacts mental health change are rare but important because they are less prone to confounding.

**Methods:**

We used data from the British Household Panel Survey to compare psychological distress in local authority renters who opted to buy their home under the UK's Right to Buy (RTB) policy versus those who continued to rent the same (social non-mover (SNM)) or a different (social mover (SM)) local authority property or who bought privately (owner mover (OM)). General Health Questionnaire (GHQ-12) scores before and after any change in tenure and/or address were compared across groups using a difference-in-difference approach.

**Results:**

Individuals who moved house (bought or rented) were younger while those who bought (the same or different house) were better off, more likely to be employed, and had higher educational qualifications. Individuals who bought their home (under RTB or privately) had lower distress scores from the outset. Individuals who moved house (bought or rented) experienced a rise in distress prior to moving that was no longer evident 1 year after the move. There was no evidence that changing tenure reduced psychological distress comparing (difference (95% CI)) average GHQ score 2 years preaddress and 1 year postaddress/tenure change in RTB vs SNM, SM, OM: −0.08 (−0.68 to 0.51), 0.16 (−0.70 to 1.01) and −0.17 (−1.28 to 0.94), respectively).

**Conclusions:**

Changing tenure under RTB did not, on average, impact psychological distress, suggesting that this status change did not change mental health.

## Introduction

It is argued that status comparison is an important contributor to health inequalities in rich countries where standards of living are said to be generally adequate for health and are therefore less of a plausible explanation for the health gradient.^1–4^ Differences in actual or perceived status may impact on health through stress-inducing social comparisons.^3^ For example, it has been suggested that people derive status from owning rather than renting their home,^5^ especially in societies, such as the UK, where owner occupation is seen as prestigious and social renting stigmatised.^6^ Thus, housing tenure, which is strongly associated with health, may have a status impact beyond associations with housing quality, neighbourhood conditions or wider socioeconomic factors that vary between those living in different tenures.^7^

A number of studies have explored the association of housing tenure and the independent relation of housing-related status with mental health.^7–9^ However, there is an inherent difficulty in identifying the impact of housing tenure per se, as those in different housing tenures are likely to differ in a broad range of health-related characteristics, raising the possibility of confounding. Longitudinal studies exploring associations between status change and health change provide more control for these between-person differences.^10^ Such analyses are rare in studies of housing tenure, but a recent Australian study explored within-person change in housing tenure and mental health and found no impact of owner occupation over-renting.^9^ However, as changes in tenure usually require housing moves, which may themselves be stressful and accompanied by major life events, it is of interest to explore the impact of tenure changes without an associated house move.

The UK's Right to Buy (RTB) policy, enacted in 1980, gave individuals who socially rented their home the statutory RTB it at reduced cost, extending the existing discretionary RTB.^11^ This policy provides an opportunity to explore changes in housing tenure not associated with physically moving. The policy has had a major impact, with over 2 million homes sold in its first 25 years, and it has also made a substantial contribution to the rise of owner occupation in the UK.^11^ Any status impact on health could therefore have major population implications.

We explored the impact of tenure change, with and without an associated house move, on changes in psychological distress in the UK, adding to the existing literature on housing tenure and health, which a recent systematic review identified as an area needing further research.^12^

## Methods

Data were drawn from the British Household Panel Survey (BHPS), a nationally representative longitudinal survey of British households with annual follow-up from 1991 to 2009.^13^ The original cohort, based on a clustered, stratified sample of 5505 households in Great Britain (excluding the north of Scotland and Northern Ireland), included 10 264 individuals aged 16 plus. In subsequent years, new participants were included if they were born to an original sample member, if they moved into an original sample household, or if an original sample member moved into a new household with one or more new people. In 1999 and 2001, additional recruitment of 1500 households from Scotland and Wales and 2000 households from Northern Ireland extended the sample to all of the UK, resulting in a total sample size of around 10 000 households.

We restricted attention to respondents who were living in a potentially eligible household for the RTB, that is, those households renting their home from the local authority (LA) in at least one data collection wave. The impact of RTB was explored by comparing psychological distress before and after any change in tenure, and therefore respondents were additionally required to have participated in at least two waves of data collection immediately following the wave in which they reported LA renting. Analyses were based on complete data ‘triplets’ at waves t−1, t and t+1, where any change in tenure occurred between waves t−1 and t. Our focus was on changes in distress 1 year after address/tenure change rather than distress measured at the same time as the address/tenure change to avoid the contemporaneous impact of changing status or house move on distress, and we therefore additionally restricted attention to respondents whose tenure and address at the third wave of interest (t+1) remained the same as that at the second (t). A similar design has been used to explore the status impacts of promotion.^10^

Respondents who did not move but whose tenure changed from ‘LA rented’ at wave t−1 to ‘owned’ (with or without a mortgage) at waves t and t+1 were classed as RTB. Three comparison groups were also defined (table 1). Respondents who did not move and whose tenure status remained the same were defined as social non-movers (SNMs), whereas those whose tenure at all waves was ‘LA rented’ but who moved were social movers (SMs), and those whose tenure changed from ‘LA rented’ to ‘owned’ and who also moved were owner movers (OMs). Using these definitions, respondents could contribute more than one data triplet to the same or different groups. Two analyses were therefore performed, the first based on all data triplets (allowing for clustering within an individual) and the second based on one triplet per person. The results from these analyses were almost identical. For ease of interpretation, we present results from the second approach with triplets selected as follows: individuals who changed their address and/or tenure (RTB, SM, OM) generally did so only once and the triplet in which this change occurred was selected (where there was more than one change, the first triplet was selected); individuals who stayed in the same LA rented property throughout (SNM) generally had a number of triplets available, and one of these was selected at random. Sensitivity analyses using different samples confirmed that results were unaffected.

**Table 1:** Classification of respondents according to tenure and address changes over three sampled waves of interest

	First sampled wave (t−1)	Second sampled wave (t)	Third sampled wave (t+1)	Classification
Tenure	Local authority rented	Owned*	Owned*	Right to buy
Address	–	Same as previous wave	Same as previous wave	
Tenure	Local authority rented	Local authority rented	Local authority rented	Social non-mover
Address	–	Same as previous wave	Same as previous wave	
Tenure	Local authority rented	Local authority rented	Local authority rented	Social mover
Address	–	Change from previous wave	Same as previous wave	
Tenure	Local authority rented	Owned*	Owned*	Owner mover
Address	–	Change from previous wave	Same as previous wave	

*With or without mortgage.

Psychological distress was measured using the 12-item version of the General Health Questionnaire (GHQ-12)^14^ ^15^ (range 0–36), with higher scores indicating higher levels of distress. Potential time-varying confounding variables at each wave included: age, marital status (married/cohabiting vs not married/cohabiting), employment status (employed/training vs unemployed/sick vs maternity leave/home carer/studying vs retired), financial status (comfortable/doing all right vs getting by vs finding it quite/very difficult), highest educational qualification (none/primary vs lower secondary vs upper secondary/postschool) and year of observation (for any period effects). Missing confounding variables were replaced, where possible, with values from the immediately preceding wave.

Analyses were based on a difference-in-difference (fixed effect) approach, where average within-person changes in GHQ score from t−1 to t+1 were compared in the RTB and comparison groups. This approach controls for stable differences between the RTB and comparison groups and any common trend over time;^16^ it is analogous in two waves of panel data to regression of change. We adjusted for the potential time-varying confounding variables between t−1 and t+1. This is important as changes in confounding variables, for example, marital status, may impact on changes in tenure^17^ and distress over the period of interest. Robust SEs were calculated, allowing for the non-independence of respondents from the same household. In order to explore the role of pre-existing differences in distress between comparison groups in more detail, additional analyses were performed on a smaller sample of respondents who were living in an LA rented accommodation at t−2, that is, 2 years before any address/tenure changes, and for whom data were available for at least four waves. That comparison groups have similar trends before the ‘intervention’ is an important consideration in difference-in-difference models.

## Results

In total, 32 380 individuals took part in at least one data collection wave. Of these, 6917 (21.4%) rented their home from the LA in at least one wave. These respondents were less qualified and less likely to be employed than those who were never LA renters. In addition, LA renters were more likely to be women, to be unmarried/not cohabiting and tended to have higher distress scores. A total of 4871 (70.4%) LA renters took part in two immediately subsequent waves and, of these, 3771 (77.4%) had complete data for GHQ and all confounding variables (21.1% had missing GHQ, 1% had missing education, 0.3% missing marital status and 0.1% each missing employment and financial status). Respondents with complete data were more likely to be older, to be women, to be married/cohabiting and were better qualified than those with incomplete data. However, GHQ scores, where available, were similar among those with and without complete data.

Characteristics of respondents in the four address/tenure groups are presented in table 2. Characteristics are measured before any address and/or tenure change (at t−1) unless stated otherwise. Almost two-thirds of LA renters had no change in address or tenure (N (%)=2475 (65.6%)). However, around 14% (N=537) lived in a house that was bought between t−1 and t, presumably under the RTB. A similar number moved to another LA rented property (N (%)=506 (13.4%)) and a smaller proportion moved to a different bought property (253 (6.7%)). Respondents who moved either to LA rented (SM) or to owned (OM) homes tended to be younger, while those who remained in their LA rented home (SNM) were older. RTB respondents were more likely to be married/cohabiting, and respondents who bought their home (RTB and OM) were better off, more likely to be employed, and had higher qualifications. Similar differences were also observed after any changes in address/tenure (not shown), with the exception of a sharp increase in OM respondents who were married/cohabiting at t+1, suggesting that, in many cases, a relationship change prompted the move.

**Table 2:** Characteristics of right to buy, social non-mover, social mover and owner mover respondents with complete data

	Right to buy (N=537)	Social non-mover (N=2475)	Social mover (N=506)	Owner mover (N=253)	p Value
*N (%)*					
Sex					
Male	237 (44.1)	1035 (41.8)	197 (38.9)	109 (43.1)	0.38
Female	300 (55.9)	1440 (58.2)	309 (61.1)	144 (56.9)	
Age					
<30	134 (25.0)	554 (22.4)	190 (37.6)	107 (42.3)	<0.001
30–59	311 (57.9)	1033 (41.7)	218 (43.1)	130 (51.4)	
60+	92 (17.1)	888 (35.9)	98 (19.4)	16 ( 6.3)	
Marital status*					
Married	355 (66.1)	1151 (46.5)	267 (52.8)	125 (49.4)	<0.001
Unmarried	182 (33.9)	1324 (53.5)	239 (47.2)	128 (50.6)	
Financial status†					
Comfortable	338 (62.9)	1040 (42.0)	186 (36.8)	154 (60.9)	<0.001
Getting by	162 (30.2)	997 (40.3)	201 (39.7)	72 (28.5)	
Difficult	37 ( 6.9)	438 (17.7)	119 (23.5)	27 (10.7)	
Employment status‡					
Employed	362 (67.4)	782 (31.6)	153 (30.2)	179 (70.8)	<0.001
Unemployed	103 (19.2)	954 (38.5)	270 (53.4)	60 (23.7)	
Retired	72 (13.4)	739 (29.9)	83 (16.4)	14 ( 5.5)	
Educational qualifications§					
None/primary	227 (42.3)	1518 (61.3)	265 (52.4)	71 (28.1)	<0.001
Secondary/above	310 (57.7)	957 (38.7)	241 (47.6)	182 (71.9)	
*Mean (SD)*					
GHQ score					
Wave t−1¶	10.6 (5.2)	12.3 (6.2)	13.4 (7.2)	11.5 (6.0)	<0.001
Wave t+1**	10.9 (5.2)	12.4 (6.2)	12.9 (6.9)	10.8 (5.4)	<0.001

*Married or cohabiting versus not.

†Comfortable or doing all right versus getting by versus finding it quite or very difficult to get by.

‡Employed or training versus unemployed, sick, maternity leave, home carer or studying versus retired.

§No or primary qualification versus secondary or higher qualification.

¶Wave immediately preceding any change in address and/or tenure.

**Wave 1 year on from any change in address and/or tenure.

GHQ, General Health Questionnaire.

There were also marked differences in GHQ distress scores between groups, before and after any address/tenure changes. Prior to any changes (at t−1), distress scores were lower among OM and particularly RTB respondents, and were highest among LA renters who moved to another LA rented property (SM). A similar pattern was observed after any address/tenure changes (at t+1), with the lowest distress scores in those who owned their current home (RTB and OM). However, while distress in those who did not move remained similar (SNM) or increased slightly (RTB) over time, there was some evidence of a reduction in distress in those who moved and particularly among those who bought their house (OM).

Table 3 presents differences in the within-person change in GHQ distress scores between t−1 and t+1 in RTB versus comparison groups. RTB versus SNM considers respondents who did not move and compares those who opted to buy with those who did not. Although distress scores at t−1 were lower in respondents who bought their home, there was no difference in the change in distress over time, that is, existing differences at t−1 were maintained at t+1. Adjustment for time-varying confounders had little impact. RTB versus SM compares respondents opting to buy their current home with those moving to another LA rented property. There was weak evidence that distress in SM respondents decreased over time in comparison with RTB respondents (difference in change (95% CI −0.70 (−1.51 to 0.12)), although the CI did not exclude 0 and the differences were somewhat attenuated by adjustment for confounders. Finally, RTB versus OM considers respondents who bought their home and compares those who bought under the RTB with those who bought privately. There was stronger evidence of a difference in the changes in distress over time, with scores decreasing by 1.01 (−2.02 to 0.01)) in OM relative to RTB respondents. Although this CI does not strictly exclude 0, it is worth noting that this comparison is based on fairly small numbers. Adjustment for confounders had a small attenuating effect.

**Table 3:** Differences (95% CI) in pre (t−1) and post (t+1) move General Health Questionnaire scores in right to buy (RTB) versus comparison groups (social non-mover (SNM), social mover (SM) and owner mover (OM)) in respondents with complete data

	N (RTB/ comparison group)	Unadjusted	Adjusted for confounding variables*
RTB vs SNM	537/2475	−0.17 (−0.71 to 0.38)	−0.26 (−0.78 to 0.26)
p Value		0.55	0.33
RTB vs SM	537/506	−0.70 (−1.51 to 0.12)	−0.52 (−1.30 to 0.26)
p Value		0.09	0.19
RTB vs OM	537/253	−1.01 (−2.02 to 0.01)	−0.84 (−1.89 to 0.21)
p Value		0.05	0.16

*Age, marital status, employment status, financial status, highest educational qualification.

**Figure 1:**
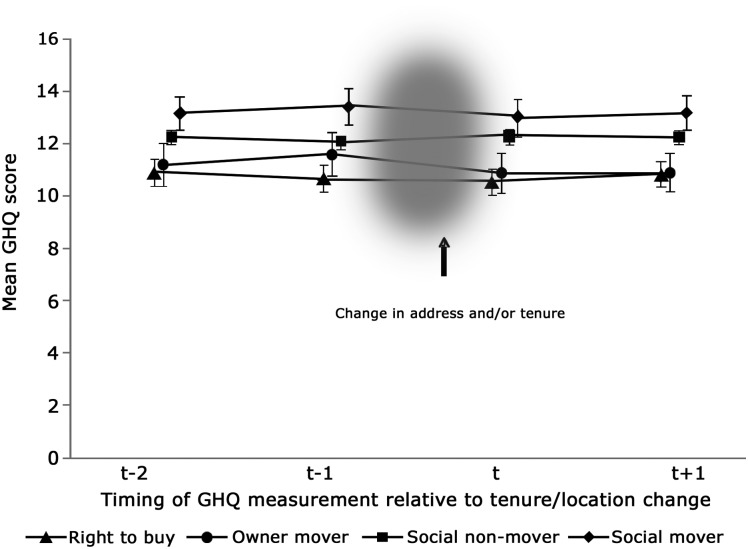
Unadjusted mean General Health Questionnaire score at t−2, t−1, t and t+1 in right to buy, social non-mover, social mover and owner mover respondents with complete data.

Analyses were repeated for those living in an LA rented accommodation 2 years prior to any address/tenure changes to explore the impact of pre-existing GHQ differences in more detail. Unadjusted mean GHQ distress scores at t−2, t−1, t and t+1 for the four address/tenure groups are shown in figure 1. Scores at waves t−1, t and t+1 were very similar to those based on the larger data set (table 1) and the previously observed differences in distress at t−1 are clearly seen in figure 1, along with decreases in distress between t−1 and t+1 in SM and, particularly, OM respondents. However, in contrast, distress scores at t−2 were very similar to those at t+1 in all four groups. This is reflected in table 4, which presents changes in GHQ score from t−2 to t+1, and shows no difference between RTB and any of the comparison groups. So while distress at all time-points differed between groups, particularly those who bought versus those who continued to rent, changes in distress *over time* were driven by a temporary rise preceding a physical change of address at t−1, which was largely resolved by t+1.

**Table 4:** Differences (95% CI) in premove (t−2) and postmove (t+1) General Health Questionnaire scores in right to buy (RTB) versus comparison groups (social non-mover (SNM), social mover (SM) and owner mover (OM)) in respondents with complete data

	N (RTB/ comparison group)	Unadjusted	Adjusted for confounding variables*
RTB vs SNM	410/2032	0.10 (−0.51 to 0.71)	−0.08 (−0.68 to 0.51)
p Value		0.74	0.79
RTB vs SM	410/400	0.10 (−0.78 to 0.97)	0.16 (−0.70 to 1.01)
p Value		0.83	0.72
RTB vs OM	410/200	−0.30 (−1.44 to 0.84)	−0.17 (−1.28 to 0.94)
p Value		0.61	0.76

*Age, marital status, employment status, financial status, highest educational qualification.

## Discussion

We used the RTB to explore the impact on psychological distress of a status change, specifically housing tenure. Our results contribute to sparse longitudinal evidence on housing tenure status and health, and should be evaluated in this context. What is notable about the present analyses is the use of the RTB, which represents a change of status separate from changes in housing or neighbourhood circumstances. Our results therefore test the theory that status per se is an important risk factor for health in rich countries, independent of any material residential changes. Of course, RTB also involves a possible change in financial circumstances, with ownership of a considerable asset transferring from the state to the individual, and we therefore included controls for changing financial security.

We found no evidence that RTB respondents’ mental health improved relative to the comparison groups; if anything, those moving house, either bought or rented, showed an improvement over RTB respondents, although this was driven by temporary premove rises in distress, possibly due to the anticipatory stress related to the forthcoming move or because stressors prompted or were relieved by the move.^18^ Other than these temporary premove peaks, distress generally remained constant over time in all groups. This is consistent with previous Australian work on tenure and mental health, using a fixed effect design,^9^ which found no impact on the mental health of owner occupation versus renting. Of note in the present analyses are the pre-existing differences between the RTB and comparison groups. Individuals who bought their home (under RTB or privately) were better off, better qualified, more likely to be employed and, most strikingly, had lower distress scores from the outset.

An overview of the literature suggested that there is mixed evidence on whether changes in other measures of status impact health.^10^ For example, previous work has identified a negative impact of promotion at work,^10^ mixed results for the winning of status awards or competitions,^19^ and gender-specific impacts only when comparing sociological measures of status to social class, education and income.^20^ It is important to recognise that status in our context is treated as an exposure independent of other markers of stratification such as income. An alternative hypothesis is that other forms of stratification affect health in part through status differentiation. For example, in a study of housing tenure, there was evidence that the advantages of tenure status were not derived from ownership versus renting per se, but rather from differences in the quality of housing and neighbourhood associated with different tenures and, ultimately, their affordability.^5^ There is good quality evidence that improving housing conditions can improve mental health,^21^ potentially through improving psychosocial conditions related to status,^8^ and so status change related to the relative improvement of material circumstances may be important for mental health. So, for example, if under RTB people improved their housing circumstances, for example, through the autonomy of ownership and personal wealth created, it could well have longer term (than our 1 year of follow-up) benefits for mental health. More generally, we have only assessed the RTB's relationship with one health outcome, limited to 1 year of follow-up and studied it only as a mechanism to leverage a housing status change independent of moving; so in no way should this study be taken as a comprehensive evaluation of the health impact of RTB. Further, we did not directly measure people's perception of their housing status, an interesting alternative approach.^22^ Such an approach would also have allowed us to unpack whether status was gained from self-recognition of the status change and/or from external recognition by peers, a difference highlighted by a reviewer. If it was more the latter, then the RTB group may not have experienced a large status change as their status change may not have been easily recognised externally.

### Strengths and limitations

BHPS is a large representative sample of households in the UK with a lengthy follow-up. Using the RTB scheme allowed us to look at the impact of changes in housing tenure independent of the related changes in address. We included controls for time-varying factors and common trends, and used three different comparison groups.

However, there are also a number of limitations. Our target population was individuals living in eligible households under the RTB and we selected LA renters in the first instance. We have no reason to believe that these respondents were not representative. However, we excluded respondents who did not take part in two additional waves, who did not remain in their home for 1 year after any move, and who did not fall into our four comparison groups, for example, those moving into private renting. This may limit the generalisability of our results, although it is reassuring that there were no differences in psychological distress at baseline among LA renters included and excluded from the analyses. Moreover, our selection process was based on the years in which data were available, and we may have missed individuals who bought their home before 1991 or in any missing years; specifically, we did not include those who used RTB in considerable numbers in the 1980s.^11^ We adjusted analyses for a range of potential time-varying confounding factors but cannot rule out the possibility of unmeasured time-varying confounding, especially as RTB is an economic-influenced choice, rather than randomly allocated, and so was taken up by more affluent tenants. In particular, we did not include time-varying housing quality as the BHPS only asked such questions from 1996, although evidence suggests that non-movers are much less likely to change housing quality than movers.^23^ In line with previous BHPS research,^24^ RTB was derived from tenure changes with no house move and may be subject to measurement error as respondents were not directly asked if they lived in an RTB house. Additionally, everyone in a household aged 16 and above could be included in the sample and some may have been quite peripheral to the decision to the RTB or move home (and thus any associated status impact). Finally, the number of individuals who opted to buy under RTB was relatively small and comparisons have limited power. That this is a limitation in such a substantial, representative cohort highlights the inherent difficulties of analyses of this type.

## Conclusions

We used the RTB as a housing tenure status change, independent of other major residential changes and explored whether a change in status per se was associated with changes in psychological distress. There were important baseline differences between those who did and did not change tenure status. However, there was no change in distress associated with changing status through the RTB scheme in the year following the tenure change, suggesting that housing status change per se is not an important driver of mental health.

**What is already known on this subject**

- Actual or perceived status, such as housing tenure, may impact on health through stress-inducing social comparisons. However, housing tenure is associated with a broad range of health-defining characteristics and, in addition, changes in tenure usually require a physical move and are often accompanied by major life changes. This makes the exploration of the impact of the status of housing tenure on health, especially mental health, particularly challenging.

**What this study adds**

- We have used the UK's Right to buy scheme, where tenure changes but the household does not move, as a change of status separate from changes in housing or neighbourhood circumstances. We explored differences in the changes in psychological distress due to changes in tenure and location. From the outset, we found that individuals who buy their home differ from those who do not, including in terms of psychological distress. Given these baseline differences, there was no impact of tenure per se and only a short-term negative impact of moving house on psychological distress. A housing status change did not impact on mental health in the year following the change.
